# The relationship between self-care ability and frailty in rural Chinese older adults: the mediating role of depressive symptoms

**DOI:** 10.3389/fpubh.2025.1713474

**Published:** 2026-01-12

**Authors:** Renjie Zhang, Luhao Liu, Jiaqi Tian, Qiyang Huai, Heng Sun, Tengfei Jiang, Lijuan Yang, Minmin Leng

**Affiliations:** 1 School of Nursing, Shandong Second Medical University, Weifang, Shandong, China; 2 Department of Nursing, Shandong Provincial Hospital Affiliated to Shandong First Medical University, Jinan, Shandong, China; 3 School of Nursing and Rehabilitation, Shandong University, Jinan, Shandong, China

**Keywords:** frailty, depressive symptoms, self-care ability, rural elderly, mediation analysis

## Abstract

**Objective:**

As the pace of population aging accelerates, rural elderly populations face multiple health challenges including depression and frailty, and their potential interactive mechanisms remain incompletely understood. To address this gap, this study investigates the relationships among depressive symptoms, frailty, and self-care capacity in rural older adults, with a specific focus on elucidating the mediating role of depressive symptoms.

**Methods:**

A convenience sampling method was used to select 5,389 rural elderly people for the Self-designed General Information Questionnaire, Frailty Phenotype, Patient Health Questionnaire-9, and Self-Care Ability Scale. Linear regression equations and self-help sampling methods were used to verify the mediating role of depressive symptoms in self-care ability and frailty.

**Results:**

Among rural older people, the prevalence of frailty was 26.5%, the prevalence of depressive symptoms was 19.5%, and the average self-care ability score was 58.91 ± 5.66. Self-care ability was negatively correlated with frailty (*r* = −0.213, *p* < 0.01) and depressive symptoms (*r* = −0.133, *p* < 0.01). Depressive symptoms were positively correlated with the degree of frailty (*r* = 0.355, *p* < 0.01).

**Conclusion:**

This study revealed a significant negative correlation between self-care ability and frailty in rural older adults, with depressive symptoms as a mediator.

## 1 Introduction

Population aging has become a major global challenge that continues to draw increasing attention from the international community. It is projected that by 2050, more than one-fifth of the global population will be 65 years of age or older (1, 2). As this trend accelerates, China's elderly population (aged 60 and above) has grown dramatically, reaching 267 million—accounting for 18.9% of the total population—making it the country with the largest elderly population globally (3). In response to the public health challenges posed by its rapidly aging population, China has elevated the achievement of healthy aging to a core objective at the national strategic level, which represents a development of significant theoretical and practical importance (4, 5). Healthy aging is defined as the process of developing and maintaining functional capacity in later life, enabling older adults to sustain quality of life and enjoy a positive state of health (6). Healthy aging involves not only safeguarding the physical health and quality of life of older adults, but also the coordinated development of multiple dimensions including emotional wellbeing and social participation (7).

Against this backdrop, frailty, which is a prevalent and complex geriatric syndrome, has emerged as a key challenge impeding the achievement of healthy aging and poses a serious threat to the overall health of older adults (8, 9). Frailty, a multidimensional clinical syndrome closely associated with aging, reflects a progressive decline in physiological reserve across multiple organ systems, leading to reduced resilience and increased vulnerability to stressors and adverse health outcomes (10, 11). Frailty not only weakens the physical function, cognitive ability and mental health of older persons but also limits their ability to take care of themselves in daily life and participate in social activities, thereby imposing substantial burdens on families and society (12–14). Evidence from major reviews confirms that frailty is not solely determined by biological aging but is also influenced by psychosocial and behavioral factors (4).

As a key behavioral factor, self-care ability, as a series of basic, continuous and purposeful human activities that individuals perform to maintain their physical and mental health, comfort and good adjustment, is a part of people's daily lives (15, 16). The relationship between self-care ability and frailty is of particular clinical importance. Declining self-care ability can hinder effective use of health resources, increase dependency, and worsen mental and physical health outcomes, thereby accelerating frailty progression (15, 17, 18). A related study revealed that the total self-care ability score of elderly hospitalized debilitated patients was at an intermediate level and that patients in the debilitated group showed a significant decrease in self-care ability compared with patients in the nondebilitated group (19).

Depressive symptoms represent another crucial factor in this complex relationship (20). Depressive symptoms affect about 15% of older adults (≥65 years) worldwide, with women bearing a greater burden (21, 22). Depressive symptoms severely affect an individual's motivation and initiative to engage in self-care practices (23). An individual's self-care ability plays a key role in the prevention and treatment of depressive symptoms. Research has shown that individuals with greater self-care competence are more likely to adopt active lifestyles, including engaging in regular physical exercise, getting adequate sleep, and practicing effective stress management, which are important factors in reducing depressive symptoms (24, 25).

A longitudinal study by Andrew et al. established a link between depression and an increased risk of frailty in older adults, and subsequent evidence further confirms a strong association between depressive symptoms and frailty, indicating that individuals with depressive symptoms are more likely to develop frailty (26, 27). Depressive symptoms increase the risk of adverse outcomes in older adults, and patients with depressive symptoms have significantly lower somatic and mental functioning as well as quality of life (28). Based on a review of the literature, we predicted that depressive symptoms mediate the relationship between self-care capacity and frailty among rural older adults.

Declining self-care ability can hinder effective use of health resources, increase dependency, and worsen mental and physical health outcomes, thereby accelerating frailty progression. However, evidence remains limited regarding how self-care ability interacts with frailty among older adults. Moreover, existing research has given limited attention to the issue of frailty among rural elderly individuals. Therefore, this study aims to explore the relationship between self-care ability and frailty in Chinese rural older adults to inform strategies for promoting healthy aging.

## 2 Methods

### 2.1 Participants

This cross-sectional study was conducted between April 2024 and June 2024, and convenience sampling was used to select study participants who met the inclusion criteria for a face–to-face survey at rural locations in Xintai City, Shandong Province. The inclusion criteria were as follows: (1) aged ≥ 60 years; (2) lived in rural areas; (3) were able to understand and complete the questionnaire; and (4) provided informed consent and voluntary participation in the study. The exclusion criteria were as follows: (1) patients with dementia; (2) those who were unable to cooperate in completing the survey; and (3) those with serious illnesses. The study was approved by the Ethics Committee of Shandong Provincial Hospital (SWYX:NO.2024-234). All participants signed an informed consent form before enrollment.

### 2.2 Survey tools

#### 2.2.1 Demographic information

A self-designed questionnaire was used, which included sex, age, BMI, marital status, education, residence status, number of children, monthly income, smoking, alcohol consumption, and chronic disease incidence.

#### 2.2.2 Frailty

Frailty phenotype (FP): FP was proposed by Freid et al. and includes five phenotypes: unexplained weight loss; self-perceived fatigue; slow walking speed; weakened grip strength; and decreased exercise. For a total score of 0–5, the higher the score is, the more severe the frailty. A score ≥3 is considered frailty, a score of 1–2 is considered prefrail, and 0 points is considered no frailty (11). The Chinese version of the scale has good reliability and validity and is applicable to the Chinese elderly population (29).

#### 2.2.3 Depressive symptoms

Patient Health Questionnaire-9 (PHQ-9): This form was developed by Kroenke et al. (30) and consists of nine items scored on a four-point Likert scale ranging from 0 to 3. A total score of < 5 is considered normal, whereas a score of 5 and above is defined as a decrease in mental competence, and the higher the total score is, the more severe the depressive symptoms (30). Liu et al. (31) applied the PHQ-9 to Chinese elderly individuals and confirmed that it has good effectiveness and reliability.

#### 2.2.4 Self-care ability

The Self-Care Ability Scale for the Elderly (SASE) was developed by O'Söderhamn in 1996 and consists of 17 items in three dimensions (Skills, Goals, and Environment) for all older adults (32). A five-point Likert scale was used, ranging from “totally disagree” to “totally agree” on a scale of 1–5, with a total score of 17–85, with higher scores indicating a greater level of self-care ability and greater potential for elderly individuals. The Chinese version of the Self-Care Ability Scale for the Elderly (SASE-CHI) is a highly effective screening tool for assessing the self-care abilities of older Chinese adults. A total score of ≤ 66 on the Chinese version of the scale indicates a low level of self-care ability. The Cronbach's alpha coefficient for this scale in this study was 0.82 (33, 34).

### 2.3 Statistical methods

Epidata 3.1 software was used for data entry. To ensure data quality, dual independent data entry and coding were performed. Normal continuous data are expressed as the means ± standard deviations; M(P25, P75) describes nonnormal numerical information; categorical data are expressed as (*n*) and percentages (%). For one-way analysis, the χ^2^ test, ANOVA, and Kruskal-Wallis test were used based on the type and distribution of the variables. We used Spearman correlation analyses to examine associations between components of self-care ability, frailty, and depressive symptoms. The mediation model was analyzed via the PROCESS macro in SPSS software developed by Hayes (35). We analyzed depression's mediating role between self-care ability and frailty using bootstrapping (IBM SPSS 23.0), with 95% CIs (excluding 0) determining significance (Hayes' method, two-tailed *p* ≤ 0.05).

## 3 Results

**Figure 1:**
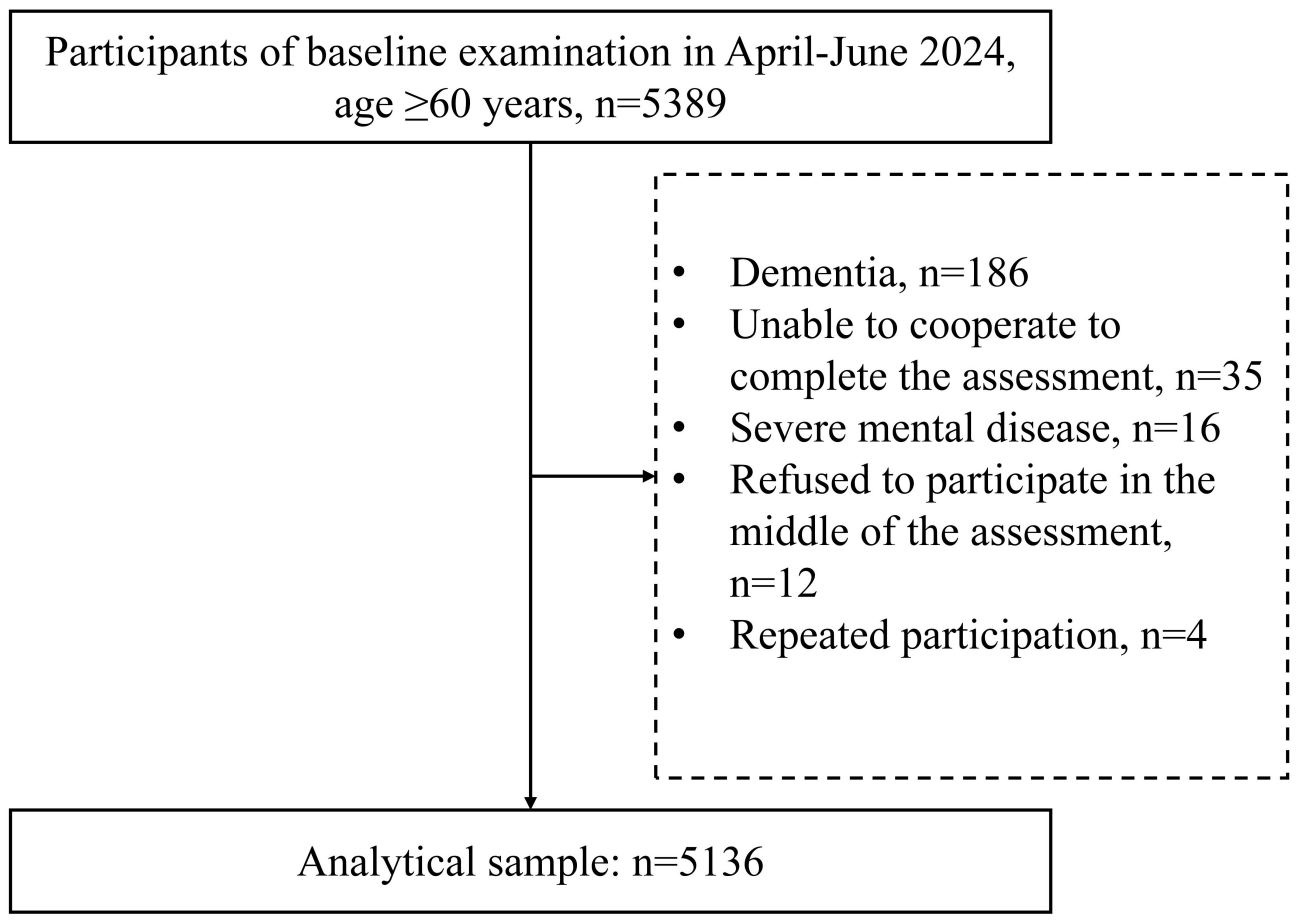
Flowchart of the study participants.

The study surveyed 5,389 rural elders, excluding 186 with dementia, 35 uncooperative, 16 with severe mental illness, 12 mid-survey refusals, and four duplicates, yielding 5,136 final participants (Figure 1).

### 3.1 Demographic characteristics

Among the 5,136 study participants, 1,867 (36.4%) were male and 3,269 (63.6%) were female; the median age was 70 (66, 75) years. The demographic information is detailed in Table 1. The overall prevalence of frailty was 26.5%, with significantly higher rates observed in women (28.2%) than in men (23.4%). The prevalence of frailty was significantly greater in the population characterized by advanced age, female sex, the absence of a spouse, low education, living alone, a high number of children, smoking, alcohol consumption, and comorbidities of chronic diseases (*p* < 0.05). The self-care competence score for the frail group (57.08 ± 6.23) was lower than the score for the nonfrail group (60.47 ± 4.82). The proportion of individuals with depressive symptoms was significantly greater in the frailty group (41%) than in the nonfrailty group (4.6%) and the prefrailty group (13.5%; *p* < 0.05).

**Table 1:** Characteristics of the study participants stratified by frailty status.

**Characteristic**	**Total**	**No-frailty**	**Prefrailty**	**Frailty**	***p*-Value**
	**(*****n*** = **5,136)**	**(*****n*** = **604)**	**(*****n*** = **3,173)**	**(*****n*** = **1,359)**	
Age (IQR)	70 (66, 75)	66 (62, 71)	70 (65, 74)	73 (69, 78)	< 0.01
**Gender (%)**					
Male	1,867 (36.4)	272 (45.0)	1,159 (36.5)	436 (32.1)	< 0.01
Female	3,269 (63.6)	332 (55.0)	2,014 (63.5)	923 (67.9)	
BMI $\left(\bar{x}\pm s\right)$	24.81 ± 3.66	24.88 ± 3.50	24.88 ± 3.70	24.60 ± 3.64	0.032
**Marital status (%)**					
Married	3,926 (76.4)	529 (87.6)	2,453 (77.3)	944 (69.5)	< 0.01
Separated	48 (0.9)	4 (0.7)	33 (1.0)	11 (0.8)	
Widowed	1,147 (22.3)	69 (11.4)	679 (21.4)	399 (29.3)	
Single	15 (0.4)	2 (0.3)	8 (0.3)	5 (0.4)	
**Education level (%)**					
Illiteracy	2,749 (53.5)	192 (31.8)	1,652 (52.1)	905 (66.6)	< 0.01
Elementary	1,259 (24.5)	161 (26.7)	806 (25.4)	292 (21.5)	
Middle	750 (14.6)	142 (23.5)	492 (15.5)	116 (8.5)	
High	344 (6.7)	95 (15.7)	206 (6.5)	43 (3.2)	
College and above	34 (0.7)	14 (2.3)	17 (0.5)	3 (0.2)	
**Living arrangement (%)**					
Live alone	1,115 (21.7)	81 (13.4)	661 (20.8)	373 (27.4)	< 0.01
Living with children	217 (4.2)	20 (3.3)	125 (3.9)	72 (5.3)	
Living with a spouse	3,804 (74.1)	503 (83.3)	2,387 (75.3)	914 (67.3)	
**Number of children (%)**					
0	21 (0.4)	3 (0.5)	10 (0.3)	8 (0.6)	< 0.01
1	399 (7.8)	88 (14.6)	232 (7.3)	79 (5.8)	
2	2,575 (50.1)	340 (56.3)	1,667 (52.6)	568 (41.8)	
≥3	2,141 (41.7)	3,173 (28.6)	1,264 (39.8)	704 (51.8)	
**Monthly income (%)**					
< 1,000	3,909 (76.1)	362 (59.9)	2,344 (73.9)	1,203 (88.5)	< 0.01
1,000−1,999	497 (9.7)	77 (12.7)	354 (11.2)	1,259 (4.9)	
2,000−3,499	302 (5.9)	64 (10.6)	201 (6.3)	750 (2.7)	
3,500−4,999	198 (3.8)	48 (7.9)	128 (4.0)	344 (1.6)	
≥5,000	230 (4.5)	53 (8.9)	146 (4.6)	34 (2.3)	
Number of chronic diseases $\left(\bar{x}\pm s\right)$	4.54 ± 1.63	4.63 ± 1.61	4.45 ± 1.61	4.71 ± 1.67	< 0.01
**Smoking (%)**					
Daily	678 (13.2)	86 (14.2)	436 (13.7)	156 (11.5)	0.024
Former	682 (13.3)	95 (15.7)	423 (13.3)	164 (12.1)	
Never	3,776 (73.5)	423 (70.0)	2,314 (72.9)	1,039 (76.5)	
**Drinking (%)**					
Daily	653 (12.7)	90 (14.9)	418 (13.2)	145 (10.7)	< 0.01
Infrequent	545 (10.6)	84 (13.9)	339 (10.7)	122 (9.0)	
Former	611 (11.9)	80 (13.2)	360 (11.3)	171 (12.6)	
Never	3,327 (64.8)	350 (57.9)	2,056 (64.8)	921 (67.8)	
**Depressive symptoms (%)**					
Yes	1,000 (19.5)	28 (4.6)	427 (13.5)	545 (40.1)	< 0.01
No	4,136 (80.5)	576 (95.4)	2,746 (86.5)	814 (59.9)	
Self-care ability $\left(\bar{x}\pm s\right)$	58.91 ± 3.66	60.47 ± 4.82	59.40 ± 5.35	57.08 ± 6.23	< 0.01

BMI, body mass index.

### 3.2 Correlations among frailty, depressive symptoms and self-care ability

Table 2 shows the Spearman's correlations for the study variables. Frailty (*r*= −0.213, *p* < 0.01) and depressive symptoms (*r* = −0.133, *p* < 0.01) were significantly and negatively correlated with self-care ability. Depressive symptoms were significantly positively correlated with frailty (*r* = 0.355, *p* < 0.01). The findings presented above collectively demonstrate a close interrelationship among self-care ability, depressive symptoms, and frailty, thereby providing crucial empirical evidence for further investigation into their underlying mechanisms.

**Table 2:** Correlations between depressive symptoms, self-care ability and frailty.

**Variable**	**Depressive symptoms**	**Self-care ability**	**Frailty**
Depressive symptoms	1		
Self-care ability	−0.133^**^	1	
Frailty	0.355^**^	−0.213^**^	1

^**^p < 0.01.

### 3.3 Mediating effects of depressive symptoms on the association between frailty and self-care ability

**Figure 2:**
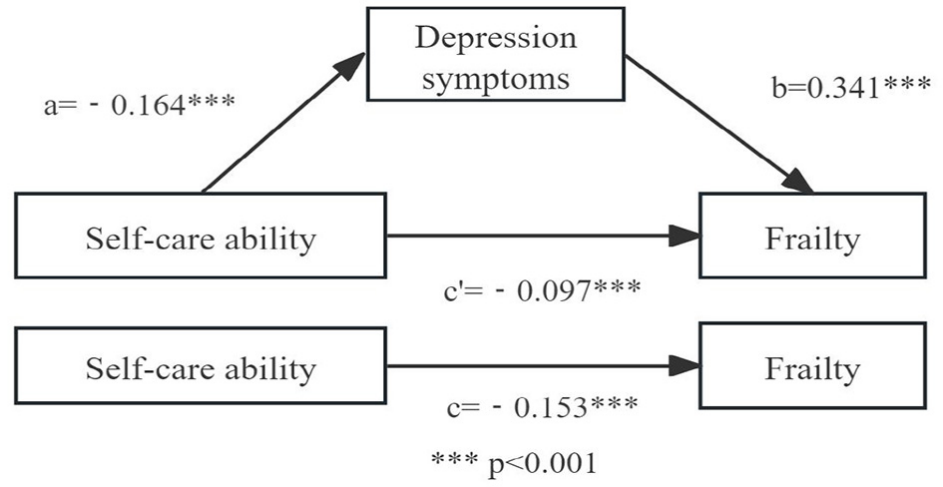
Mediating role test model diagram. ***p < 0.001.

Table 3 presents the results of the mediation analysis examining the relationships among the variables. The total effect of self-care ability on frailty was found to be significant (β = −0.153, *p* < 0.01) in Model 1. In Model 2, self-care ability significantly predicted depressive symptoms (β = −0.164, *p* < 0.01). Model 3 showed that both self-care ability (β = −0.097, *p* < 0.01) and depressive symptoms (β = 0.341, *p* < 0.01) had significant effects on frailty. Bootstrap analysis further confirmed a significant indirect effect of self-care ability on frailty through depressive symptoms, with an estimate of −0.098 and a 95% confidence interval excluding zero (−0.122 to −0.073; Table 4). These results support the presence of partial mediation by depressive symptoms in the relationship between self-care ability and frailty. The final mediation model is shown in Figure 2.

**Table 3:** Test results of the mediating role via the distribution regression method.

**Model**	**Model 1**		**Model 2**		**Model 3**	
**Variable**	**Frailty**		**Depressive symptoms**		**Frailty**	
Notation	β	*t*	β	*t*	β	*t*
Self-care ability	–0.153^**^	–11.657	−0.164^**^	−11.740	−0.097^**^	−7.826
Depressive symptoms					0.341^**^	27.938
*R* ^2^	0.178		0.065		0.287	
*F*	92.570^**^		29.725^**^		158.494^**^	

^**^p < 0.01 Gender, age, BMI, education, marital status, residence, number of children, monthly income, number of chronic diseases, smoking, and alcohol consumption were controlled.

**Table 4:** Bootstrap test results.

**Effect relation**	**Effect value**	**SE**	**LLCI**	**ULCI**	**Proportion of effect**
Total effect	−0.153	0.013	−0.179	−0.128	
Direct effect	−0.097	0.012	−0.122	−0.073	63.4%
Indirect effect	−0.056	0.007	−0.069	−0.042	36.6%

Gender, age, BMI, education, marital status, residence, number of children, monthly income, number of chronic diseases, smoking, and alcohol consumption were controlled.

## 4 Discussion

This study examined the relationship between self-care ability, depressive symptoms, and frailty among 5,136 older adults living in rural China. The overall prevalence of frailty was 26.5%, with higher rates among women, older participants, and those with lower education and income levels. In addition, lower self-care ability was significantly associated with greater frailty, and depressive symptoms played a mediating role in this relationship.

### 4.1 Comparison of different characterizations of frailty

There were significant differences (*p* < 0.05) in sex, age, marital status, years of education, number of children, residential status, monthly income, smoking status, and alcohol consumption across the different levels of frailty. Based on the results of previous studies, frailty was confirmed to be associated with older age, lower BMI, being female, living alone, having multiple chronic diseases, smoking status, and alcohol consumption status (36). Specifically, women exhibit a higher susceptibility to frailty, a phenomenon associated not only with biological factors such as hormonal changes and reduced muscle mass, but also with socioeconomic factors including limited access to resources and social isolation (37, 38). This gender disparity is further compounded by cultural expectations. Under the influence of traditional Chinese cultural norms, elderly women are often expected to demonstrate a strong tendency toward self-sacrifice, a social expectation that may cause them to overlook their personal health needs and thereby heighten their vulnerability to frailty (39, 40).

Beyond gender and cultural influences, socioeconomic factors also play a critical role. Findings from a longitudinal study conducted in Italy align with those of this research, jointly indicating a significant association between lower educational attainment and an increased risk of frailty among older adults (41). This protective effect is achieved through the lifelong accumulation of cognitive, economic, behavioral, and psychological advantages, which collectively delay the depletion of physiological reserves and mitigate frailty (42). Social connection and marital status are also critical determinants of frailty. Living alone, being unmarried, or experiencing widowhood may accelerate frailty progression in older adults through three key pathways: reduced social support, heightened loneliness and depressive symptoms, and the development of unhealthy lifestyle behaviors (43–45). A systematic review corroborates the findings of this study, indicating that higher household income is associated with a lower risk of frailty in older adults by facilitating access to healthcare, adequate nutrition, and healthier behaviors (46).

In addition to the factors above, this study also observed that the number of children may affect frailty in elderly individuals. However, the nature of this relationship appears complex and context-dependent. Antczak's (47) study revealed that the risk of poorer health for parents with more children was observed in 15 countries, but in some countries, fewer children predicted poorer health. Given this complex relationship, further studies could be conducted in the future to explore the relationship between the number of children and frailty.

### 4.2 Status of self-care ability, frailty and depressive symptoms

The prevalence of depression among older adults in rural China was 19.5% in this study, closely aligned with the global rate of 19.2% reported by Jalali et al. (48). Rural elderly participants in this study exhibited a mean SASE score of 58.91 ± 3.66, with 92.6% demonstrating inadequate self-care capacity. These values were significantly lower than those observed in community-dwelling elderly individuals, who recorded a mean SASE score of 62.43 ± 7.33 and an inadequate self-care rate of 73.09% (49). The prevalence of frailty among rural elderly individuals in this study was 26.5%, which is significantly higher than the 13.6% observed in urban community populations and the national average, highlighting a distinct health disparity between rural and urban settings (50, 51). Rural residents face barriers to healthcare access and utilization due to geographic isolation and economic constraints, with both resource availability and usage rates lower than those of urban populations (52). Based on these findings, it is evident that rural older adults in China experience a disproportionately high burden of depression, inadequate self-care capacity, and frailty compared to their urban counterparts, highlighting the urgent need for targeted health interventions in rural areas.

### 4.3 Correlation among self-care ability, frailty and depressive symptoms

A systematic review confirmed that depressive symptoms are significantly associated with frailty onset and progression in older adults, while intervention evidence indicates that enhancing self-care confidence can mitigate depression's adverse effects on physical health (27, 53). Consistent with this, our study found depressive symptoms significantly correlated with reduced self-care capacity and higher frailty prevalence (both *p* < 0.05). Spearman's correlation analysis revealed a significant negative correlation between self-care ability and frailty severity, suggesting diminished self-care capacity may heighten frailty risk—a finding corroborated by an Iranian study involving elderly patients (54). Further supporting this relationship, deteriorating self-care capacity impairs physical, functional, and psychosocial health in older adults, manifesting as falls, hospitalizations, weight loss, and limitations in activities of daily living, all of which accelerate frailty progression (55, 56). Notably, a significant negative correlation was observed between PHQ-9 and SASE scores (*r* = −0.133). A systematic review substantiates this perspective, which demonstrated that enhancing self-care confidence can effectively mitigate the impact of depression on health outcomes in chronically ill patients (57). Together with existing literature, this study has collectively confirmed the close intrinsic relationships among depressive symptoms, self-care capacity, and frailty.

### 4.4 Mediating role of level of depressive symptoms in self-care ability and frailty

The mediation analysis revealed that depressive symptoms partly explained the association between low self-care ability and higher frailty levels, suggesting that psychological wellbeing may act as a pathway through which behavioral and functional factors influence physical decline. Specifically, facing declining self-care capacity, older adults require ongoing physical, psychological, and social adjustments to adapt to functional and environmental changes. In depressed older adults, diminished self-care capacity undermines self-confidence and perceived control, impairing resource mobilization. Consequently, depressive symptoms may foster helplessness, potentially accelerating frailty progression. In contrast, healthy older adults generally exhibit greater vitality and maintain better physical and mental states. Therefore, interventions that enhance self-care ability may therefore not only promote independence but also mitigate depression and subsequent frailty progression.

### 4.5 Implications for practice

These findings highlight the need for integrated interventions that address both physical and psychological components of aging, particularly in rural areas with limited resources. To promote healthy aging among rural elderly populations, coordinated actions are recommended at both institutional and societal levels. Institutionally, healthcare and elderly care resources should be integrated and their allocation optimized to enhance service coverage and efficiency. Societally, family emotional bonds need strengthening through regular contact with elderly relatives, along with implementing tailored exercise programs to foster regular physical activity habits in this population (58). Comprehensive intervention programs designed to enhance self-care capabilities, promote physical activity, and provide emotional support can effectively alleviate frailty among rural elderly populations, improve their overall quality of life, and thereby advance the process of healthy aging.

### 4.6 Strengths and limitations

This study has several strengths. First, the study is underpinned by a large-scale, high-quality dataset independently collected by the research team, providing a solid foundation for the accuracy and reliability of the subsequent analyses. Secondly, this study focuses on the rural elderly population, thereby addressing the insufficient attention, due to various constraints, paid to this group in previous research. Furthermore, this study introduces the relatively novel concept of “self-care ability” to the field of rural elderly health research, offering a fresh lens through which to view health issues in this population.

This study has several limitations. First, its cross-sectional design precludes causal inferences between self-care ability, depressive symptoms, and frailty. Second, the depressive symptoms and self-care ability variables in this study were subjectively reported by the participants and may be biased. Finally, the use of convenience sampling in one region may limit generalizability to other populations. Future longitudinal studies are needed to confirm the mediating role of depression and to explore whether interventions targeting self-care ability can reduce frailty incidence.

## 5 Conclusions

In summary, this study provides evidence that low self-care ability is strongly associated with frailty among rural older adults, and that depressive symptoms serve as an important mediator in this relationship. These results underscore the importance of promoting both psychological wellbeing and self-care competence as part of comprehensive strategies to prevent frailty in aging populations.

## Data Availability

The original contributions presented in the study are included in the article, further inquiries can be directed to the corresponding author.
